# Short-Term Prognostic Factors in Hospitalized Herpes Zoster Patients and Its Associated Cerebro-Cardiovascular Events: A Nationwide Retrospective Cohort in Japan

**DOI:** 10.3389/fmed.2022.843809

**Published:** 2022-03-04

**Authors:** Yuichi Ishikawa, Kazuhisa Nakano, Kei Tokutsu, Shingo Nakayamada, Shinya Matsuda, Kiyohide Fushimi, Yoshiya Tanaka

**Affiliations:** ^1^The First Department of Internal Medicine, School of Medicine, University of Occupational and Environmental Health, Japan, Kitakyushu, Japan; ^2^Sato Clinic, Ebisu, Shibuya-ku, Tokyo, Japan; ^3^Department of Rheumatology, Kawasaki Medical School, Kurashiki, Japan; ^4^Department of Preventive Medicine and Community Health, University of Occupational and Environmental Health, Kitakyushu, Japan; ^5^Department of Health Policy and Informatics, Tokyo Medical and Dental University Graduate School, Tokyo, Japan

**Keywords:** herpes zoster (HZ), cerebrovascular event, glucocorticoids, DPC (Diagnosis Procedure Combination), nationwide administrative database, cardiovascular event

## Abstract

**Background:**

Short-term mortality and incidence of cerebrovascular and cardiovascular events (C-CVE) during hospitalization of patients with severe herpes zoster (HZ) have not been sufficiently investigated. We aimed to investigate short-term prognosis and incidence of C-CVE associated with HZ in hospitalized patients.

**Methods:**

This retrospective cohort study from April 2016 to March 2018 included HZ inpatient cases selected from the Diagnosis Procedure Combination database—a Japanese nationwide inpatient database. HZ and C-CVE were diagnosed based on the 10^th^ revision of the International Classification of Diseases and Injuries codes. The definition of primary exposure was that treatments were initiated within 7 days of admission, and antivirals were administered for ≥7 days. Main Outcomes were in-hospital deaths and C-CVE onset after hospitalization.

**Results:**

Among 16,811,501 in-hospital cases registered from 1,208 hospitals, 29,054 cases with HZ were enrolled. The median age was 71.0 years, 15,202 cases (52.3%) were female, and the HZ types were the central nervous system (n=9,034), disseminated (n=3,051), and ophthalmicus (n=1,069) types. There were 301 (1.0%) in-hospital deaths and 385 (1.3%) post-hospitalization onset of C-CVE. The 30-day in-hospital survival rates with or without underlying disease were 96.8% and 98.5%, respectively. Age ≥75 years (hazard ratio [HR], 2.18; 95% confidence interval [CI], 1.55–3.05), liver cirrhosis or hepatic failure (HR, 5.93; 95% CI, 2.16–16.27), chronic kidney disease (HR, 1.82; 95% CI, 1.24–2.68), heart failure (HR, 1.65; 95% CI, 1.22–2.24), and old cerebrovascular events (HR, 1.92; 95% CI, 1.10–3.34) were associated with poor short-term prognosis. Age ≥75 years (odds ratio [OR], 1.70; 95% CI, 1.29–2.24), diabetes (OR, 1.50; 95% CI, 1.19–1.89), dyslipidemia (OR, 1.95; 95% CI, 1.51–2.51), hyperuricemia (OR, 1.63; 95% CI, 1.18–2.27), hypertension (OR, 1.76; 95% CI, 1.40–2.20), heart failure (OR, 1.84; 95% CI, 1.32–2.55), and glucocorticoid administration (OR, 1.59; 95% CI, 1.25–2.01) were associated with increased risks for in-hospital C-CVE onset.

**Conclusions:**

The underlying diseases that could influence the short-term mortality of severe HZ were identified. Glucocorticoid is a possible risk factor for the in-hospital onset of C-CVE after severe HZ development.

## Introduction

Herpes zoster (HZ) is an infection caused by the varicella-zoster virus (VZV). The risk of onset and severity increases in the elderly and in patients with underlying diseases that can lead to immunosuppression, such as connective tissue diseases (CTD) and malignancies (1–5). The incidence of HZ is increasing due to an aging society and the development of immunosuppressive therapy for autoimmune diseases, including CTD, and the occurrence of severe HZ may increase (6–8). Compared to other races, the incidence of HZ is higher in Japanese rheumatoid arthritis (RA) patients treated with a Janus kinase inhibitor (JAK-i), and the reasons have received attention (9, 10). Although it has been reported that mortality increases after the onset of HZ, the prognosis after the onset of HZ remains unclear due to substantial differences in survival rates among previous studies (11, 12). Moreover, information on the short-term prognosis of severe HZ and poor prognostic factors is limited. HZ is caused by various complications, including cerebrovascular and cardiovascular events (C-CVE) such as stroke and ischemic heart disease (IHD) (13). Reactivation of VZV can cause vasculitis, which can result in stroke and IHD (13, 14). The risk of developing C-CVE increases after HZ, and the risk of C-CVE may increase just after HZ onset (13, 15–18). There have been a few studies that have focused on patients hospitalized with severe HZ, which increases the incidence of death or C-CVE onset, and there is a paucity of studies that have examined short-term prognostic factors, the frequency of HZ-related C-CVE during hospitalization, and risk factors for developing C-CVE. Severe HZ increases the burden of medical costs for treating complications and sequelae (19), and the investigation of risk factors for severity and severe complications may be important public health information for taking preventive measures, including appropriate vaccination strategies against the onset and severe complications. We focused on patients with severe HZ that required hospitalization and investigated the short-term prognosis and prognostic factors and the incidence of C-CVE onset and risk factors of C-CVE onset after hospitalization for HZ treatment using the national administrative inpatient database in Japan.

## Materials and Methods

### Study Design

This was a retrospective cohort study using the Diagnosis Procedure Combination (DPC) database which is a nationwide inpatient database in Japan.

### Setting

Data were collected by the DPC research group, funded by the Ministry of Health, Labour, and Welfare, Japan. In the study period, 1,208 hospitals participated in the survey of the DPC research group and provided their data for research purpose (20). The database contains patient information and detailed procedures for the Japanese national insurance system (21, 22). Patient information recorded in the DPC database includes diagnosis based on the 10^th^ revision of the International Classification of Diseases and Injuries (ICD-10) codes at the time of admission, comorbidities, and complications after admission. The DPC database also contains information on administered drugs, blood products used, and outcomes at discharge (23, 24).

### Participant Selection

**Figure 1:**
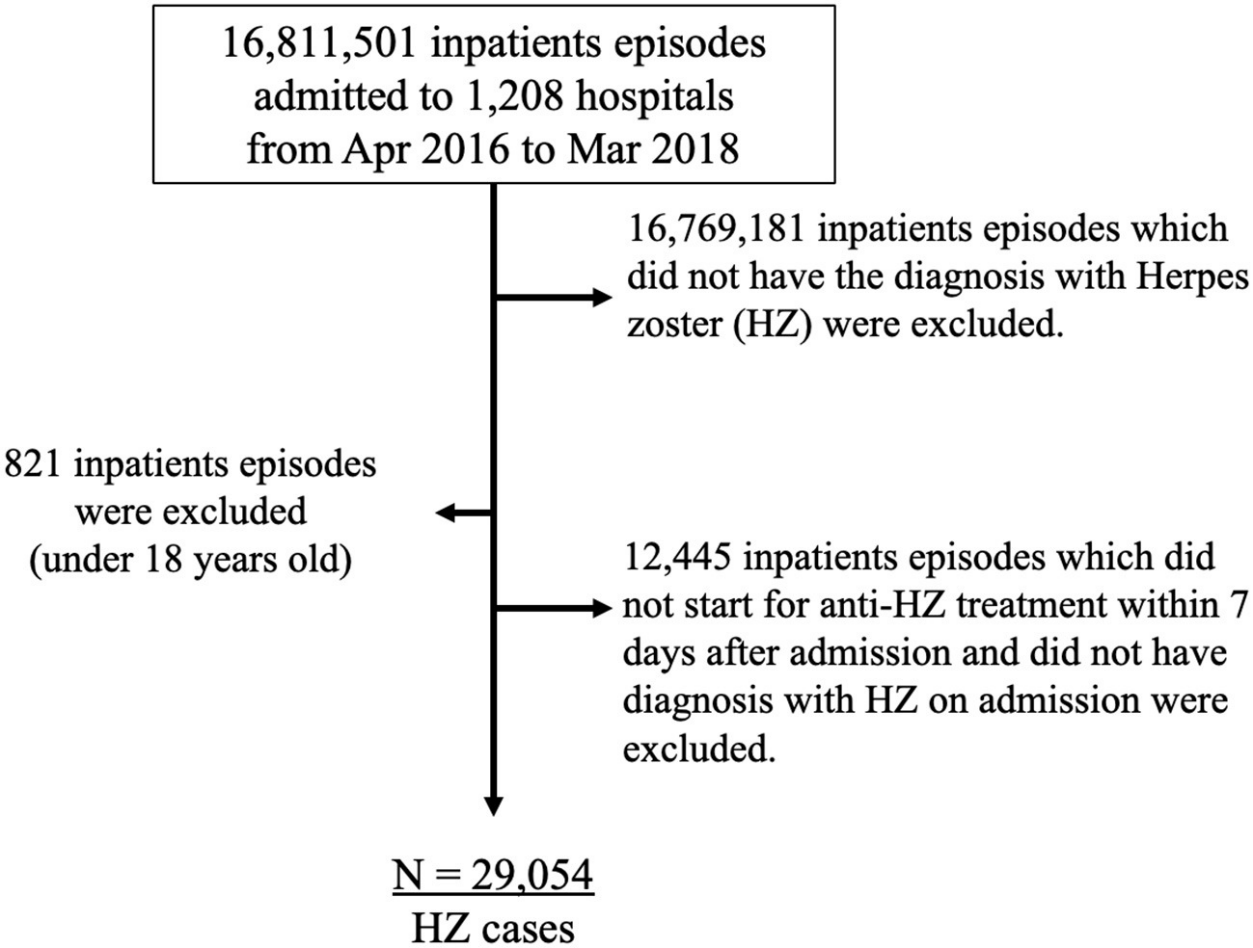
Flowchart of eligible and ineligible participants. HZ, herpes zoster.

Cases were enrolled from April 1, 2016 to March 31, 2018. First, 41,499 in-hospital cases over 18 years old who have a diagnosis of HZ and treated with HZ-specific antivirals (acyclovir, famciclovir, valaciclovir, amenamevir, or vidarabine) for 7 days or more were extracted from the DPC database among a total of 16,811,501 inpatient cases from 1,208 hospitals in the DPC database. Second, of the 41,499 cases, 12,445 cases that did not start a specific antiviral treatment within 7 days after admission and did not have a diagnosis of HZ on admission were excluded. After the exclusion, there were a total of 29,054 cases diagnosed with HZ included in the study (Figure 1).

### Definition of HZ and Underlying Diseases

The principal diagnosis of HZ was based on the ICD-10 code B02. The four types of HZ were classified as follows: central nervous system (CNS) (B02.0, B02.1), disseminated (B02.7), ophthalmicus (B02.3), and others (B02.8 and B02.9). It was expected that using the ICD-10 codes alone would identify patients who were not treated for HZ as well as those suspected with HZ who initiated treatment. To ensure robust diagnosis, we enrolled cases who received HZ-specific antivirals for at least 7 days, the standard duration of HZ treatment (25, 26). The principal diagnosis of underlying diseases was recorded using the following ICD-10 codes: malignancies, C00–C98; solid cancer, C00–C80, C97; malignant lymphoma and hematopoietic malignancies, C81–C96; human immunodeficiency virus disease, B20–B24; immunodeficiencies, D80–D84; Transplanted organ and tissue status, Z94; disorders of the thyroid gland, E05.1, E06.3; diabetes mellitus (DM); E10–E14; dyslipidemia, E78; hyperuricemia, E79.0, M10; depressive disorder, F31–F34; demyelinating diseases, G35–G37; chronic obstructive pulmonary disease (COPD), J44; asthma, J45; interstitial lung disease (ILD), J84, J99.0, J99.1; hypertension (HT) and HT-related diseases, I10–I15; heart failure (HF), I11.0, I13.0, I50, I97.1; chronic ischemic heart disease (cIHD), I25; sequelae of cerebrovascular disease (CVD), I69; inflammatory bowel diseases, K50 and K51; autoimmune hepatitis, K75.4; cirrhosis and hepatic failure, K70.3, K70.4, K71.7, K72, K74; chronic viral hepatitis, B18; CTD, L40.5, M05–M07, M30–M35, M45, M94.1 (^*^M07.4–M07.6, M30.2, M30.3, M31.5, M33.0, M34.2, and M35.3–M35.7 were excluded), RA; M05–M06, M31.5 (^*^M06.1 was excluded); systemic vasculitis, M30, M31 (^*^M30.2, M30.3, M31.5 were excluded); systemic lupus erythematosus, M32; others, L40.5, M06.1, M07, M33–35, M94.1 (^*^M07.4–M07.6, M33.0, M34.2, and M35.3–M35.7 were excluded); chronic kidney disease (CKD), N18; glomerular diseases, N00, N01, N03–N05, and N08.

### Study Outcomes

The primary outcome was overall in-hospital survival at 30 and 60 days after the initiation of treatment for HZ. The secondary outcome was in-hospital C-CVE onset after admission for HZ treatment. The diagnosis of C-CVE was based on the ICD-10 codes as follows: cerebrovascular diseases (I60–I67) and cardiovascular diseases (I20–I24). The definition of the secondary outcome was extracted from the post-hospitalization onset of secondary diseases recorded in the DPC database. The study also aimed to investigate the prognostic factors associated with in-hospital mortality and risk factors for C-CVE onset associated with HZ after admission.

### Statistical Analysis

Categorical variables are presented as numbers (%), and continuous variables are presented as medians with interquartile ranges (IQR) or numbers with percentages (%). An independent sample, the Mann–Whitney test, was employed to evaluate non-normally distributed data for comparison between the two groups. Classification data number (percentage) were aggregated. Chi-square or Fisher's exact test was performed. The log-rank test was used to compare the survival rates among the groups. Univariable Cox regression analysis and logistic regression analysis were used to screen for potential confounders associated with in-hospital mortality and in-hospital C-CVE onset after admission for HZ treatment. Associations among covariates and risk of in-hospital mortality were evaluated using multivariable Cox proportional hazards regression analysis, and associations between covariates and risk of in-hospital C-CVE onset after admission for HZ treatment were evaluated using multivariable logistic regression analysis. Hazard ratios (HRs) and odds ratios (ORs) with 95% confidence intervals (CIs) were determined after adjusting for potential confounders. Multivariable Cox regression analysis and logistic regression analysis were used to evaluate independent risk factors for in-hospital mortality and in-hospital C-CVE onset after admission for HZ treatment. The underlying diseases and confounding factors that are risk factors for HZ reported in previous studies were preferentially selected as explanatory variables for multivariable analysis using the Cox proportional hazards regression (2, 27).

Previously reported risk factors for C-CVE were preferentially selected as explanatory variables for multivariable logistic regression analysis (28, 29). We assumed that continuous variables (body mass index (BMI) and Brinkman index) were missing at random. Variables with missing values were not included in the multivariable analysis because obesity (BMI ≥ 25) and smoking history could be considered not to be confounding factors based on the results of univariable analysis. All tests were two-tailed, and the statistical significance was set at *p* < 0.05. All statistical analyses were performed using the R software package (version 4.0.0, R Foundation) (30).

## Results

### Patient Characteristics

Baseline characteristics of the study cohort are presented in Table 1. The median age was 71 years, and 15,350 cases (52.8%) were women. The cohort comprised 9,103 (31.3%), 8,095 (27.9%), and 11,856 (40.8%) cases aged 18–64 years, 65–74 years (pre-old age), and ≥75 years (old age) of age, categories based on the Japanese Gerontological Society and the Japan Geriatrics Society (31). A total of 17,973 (51.9%) cases had underlying diseases. Glucocorticoid (GC) was administered during hospitalization in 9,550 (32.9%) cases. The median length of hospital stay after the initiation of HZ treatment was 8 days. Based on the severity of HZ, most cases (21,061 cases [72.5%]) were treated with intravenous antivirals (mostly acyclovir). The median length of antiviral administration was 8 days.

**Table 1:** Patient characteristics.

	***N* (%)**
**Total number of cases**	29,054
**Age**	
Year, Median (IQR)	71 [61, 80]
18–64 (%)	9,103 (31.3)
65–74 (%)	8,095 (27.9)
75- (%)	11,856 (40.8)
**Gender**	
Male (%)	13,704 (47.2)
Female (%)	15,350 (52.8)
**Smoking**	
Brinkman index, Median (IQR)	0 [0, 50]
Smoking history (%)	6,489 (22.3)
Missing data	3,180 (10.9)
**Body mass index (BMI)**	
Median, IQR	17.8 [16.3, 19.5]
−18.4 (%)	16,724 (57.6)
18.5–24.9 (%)	10,989 (37.8)
25.0- (%)	142 (0.5)
Missing data	1,199 (4.1)
**Length of hospital stay**	
Days, Median (IQR)	9 [8, 13]
**Types of herpes zoster**	
Central nervous system (%)	9,034 (31.1)
Disseminated (%)	3,051 (10.5)
Ophthalmicus (%)	1,069 (3.7)
Others (%)	19,905 (68.5)
**Underlying disease**	
With underlying disease (%)	17,973 (61.9)
Without underlying disease (%)	11,081 (38.1)
**Malignant diseases**	
All malignancies (%)	6,882 (23.7)
Solid cancer (%)	2,283 (7.9)
Malignant lymphoma and hematopoietic malignancies (%)	4,804 (16.5)
**Autoimmune diseases**	
Connective tissue diseases (CTD) (%)	1,492 (5.1)
Rheumatoid arthritis (%)	757 (2.6)
Systemic lupus erythematosus (%)	280 (1.0)
Systemic vasculitis (%)	132 (0.5)
Other CTD (%)	426 (1.5)
Demyelinating diseases (%)	52 (0.2)
**Immune disorder**	
Human immunodeficiency virus infection (%)	75 (0.3)
Transplanted organ and tissue status (%)	281 (1.0)
Immunodeficiencies (%)	553 (1.9)
**Gastrointestinal and liver diseases**	
Inflammatory bowel diseases (%)	97 (0.3)
Autoimmune hepatitis (%)	28 (0.1)
Chronic viral hepatitis (%)	405 (1.4)
Liver cirrhosis and hepatic failure (%)	33 (0.1)
**Renal diseases**	
Chronic kidney disease (%)	613 (2.1)
Glomerulonephritis (%)	184 (0.6)
**Endocrine and metabolic diseases**	
Diabetes mellitus (%)	4,849 (16.7)
Dyslipidemia (%)	3,101 (10.7)
Hyperuricemia (%)	1,509 (5.2)
Disorders of thyroid gland (%)	47 (0.2)
**Respiratory diseases**	
Asthma (%)	610 (2.1)
Chronic obstructive pulmonary disease (%)	156 (0.5)
Interstitial lung disease (%)	307 (1.1)
**Cerebrovascular and cardiovascular diseases**	
Hypertension (HT) and HT related diseases (%)	6,408 (22.1)
Heart failure (%)	1,301 (4.5)
Chronic ischemic heart disease (cIHD) and/or Sequelae of cerebrovascular disease (CVD) (%)	1,205 (4.1)
cIHD (%)	543 (1.9)
Sequelae of CVD (%)	191 (0.7)
**Psychiatric diseases**	
Depressive disorder (%)	950 (3.3)
**Anti-herpes zoster treatment**	
Prescription days of antivirals, days, Median (IQR)	8 [7, 8]
Oral drug monotherapy (%)	5,957 (20.5)
Acyclovir (ACV) (%)	3,202 (11.0)
Valaciclovir (VCV) (%)	2,299 (7.9)
Famciclovir (FCV) (%)	328 (1.1)
Amenamevir (ANV) (%)	26 (0.1)
Intravenous monotherapy (%)	21,404 (73.7)
ACV (%)	21,349 (73.5)
Vidarabine (VDB) (%)	55 (0.2)
Combination of oral and intravenous drugs (%)	2,036 (7.0)
Oral and intravenous ACV (%)	209 (0.7)
ACV and VDB (%)	1 (0.0)
VCV and ACV (%)	1,000 (3.4)
VCV and VDB (%)	0 (0.0)
FCV and ACV (%)	292 (1.0)
FCV and VDB (%)	2 (0.0)
ANV and ACV (%)	27 (0.1)
ANV and VDB (%)	1 (0.0)
**Medications administered during hospitalization**	
Glucocorticoids (%)	9,550 (32.9)
Albumin preparations (%)	245 (0.8)
Globulin preparations (%)	632 (2.2)

*ACV, acyclovir; ANV, Amenamevir; BMI, Body mass index; C-CVE, Cerebro-cardiovascular events; cIHD, Chronic ischemic heart disease; CTD, Connective tissue diseases; CVD, Cerebrovascular disease; FCV, Famciclovir; HT, Hypertension; HZ, Herpes zoster; IHD, Ischemic heart disease; VCV, Valaciclovir; VDB, Vidarabine*.

### Study Outcomes

#### Survival Rates and Prognostic Factors

**Figure 2:**
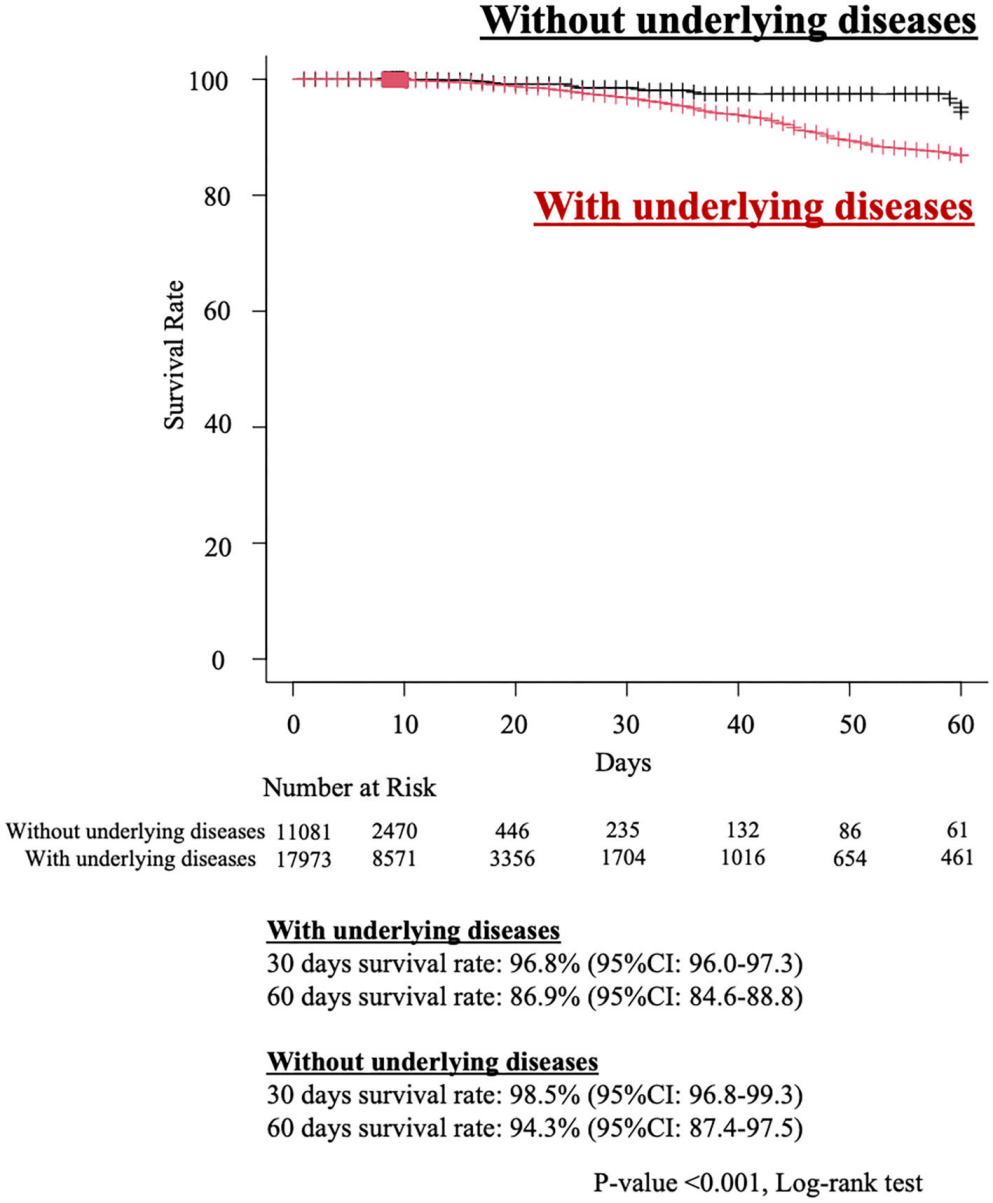
Estimated Kaplan–Meier overall survival curve of herpes zoster patients with or without underlying diseases. 95% CI, 95 percent confidence interval.

**Figure 3:**
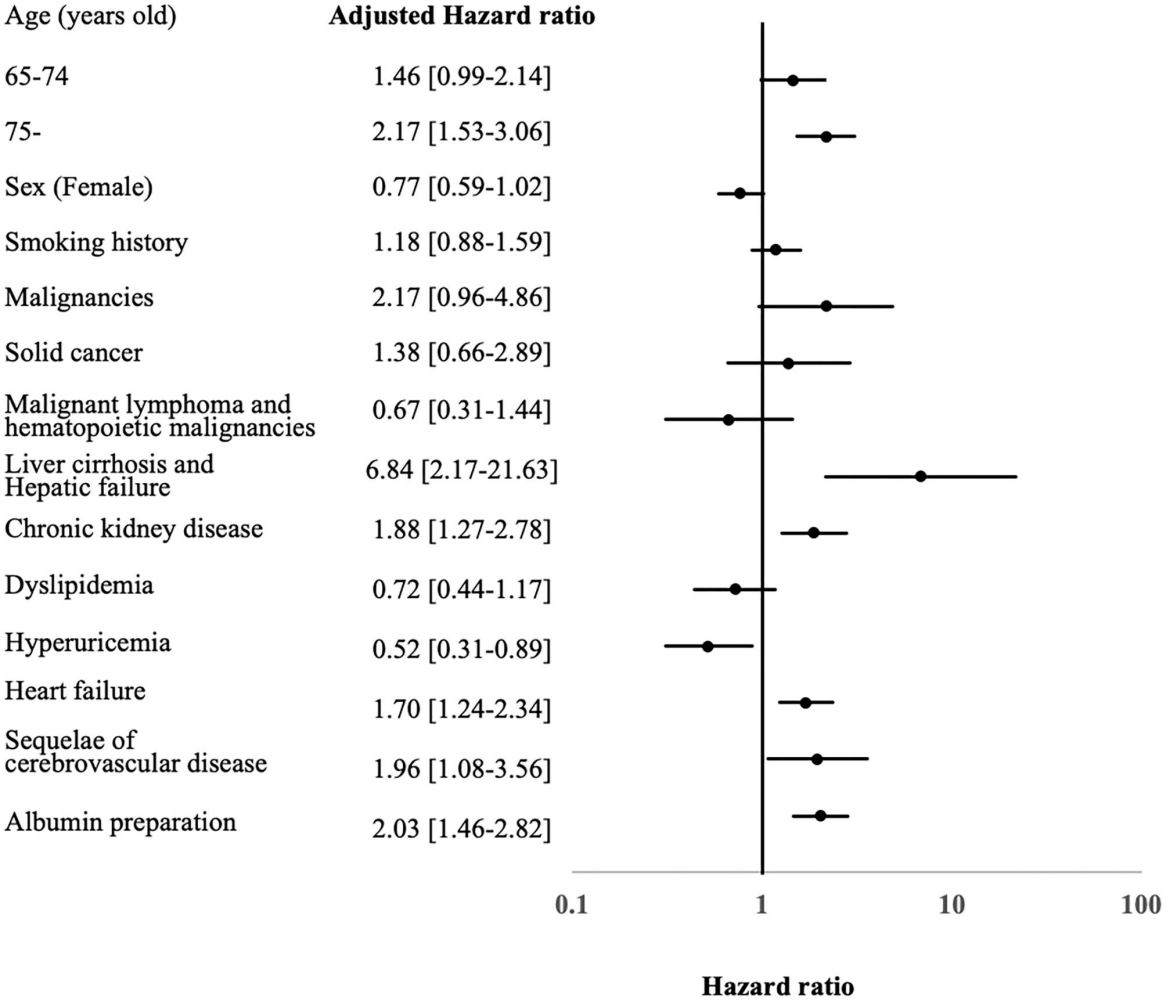
Predictors for poor prognosis of hospitalized herpes zoster cases. aHR, adjusted hazard ratio; C-CVE, cerebro-cardiovascular events; 95% CI, 95 percent confidence interval.

There were 307 in-hospital deaths (1.1%) in the study cohort. The overall survival rates at 30 and 60 days were 97.0% and 87.7%, respectively. The estimated 30- and 60-day survival rates after the start of HZ treatment for the groups with or without underlying disease were 96.8% (95% confidence interval [CI], 96.0–97.3)/86.9% (95% CI, 84.6–88.8), and 98.5% (95% CI, 96.8–99.3) and 94.3% (95% CI, 87.4–97.5), respectively (p<0.001) (Figure 2). The 30- and 60- day survival rates for each type of HZ are as follows: overall (30-day in-hospital survival rate, 97.0%; [95% CI, 96.3–97.5] and 60-day in-hospital survival rate, 87.7%; [95% CI, 85.7–89.5]), CNS (30-day in-hospital survival rate, 98.0%; [95% CI, 96.7–97.3]) and 60-day in-hospital survival rate, 88.3%; [95% CI, 85.3–89.5]), disseminated (30-day in-hospital survival rate, 96.6%; [95% CI, 93.4–98.3] and 60-day in-hospital survival rate, 85.3%; [95% CI, 75.6–91.3]), ophthalmicus (30-day in-hospital survival rate, 100%; [95% CI, NA–NA] and 60-day in-hospital survival rate, 100%; [95% CI, NA–NA]), and other (30-day in-hospital survival rate, 96.9%; [95% CI, 96.2–97.5 and 60-day in-hospital survival rate, 88.0%; [95% CI, 85.7–89.9]). The baseline characteristics of the survivor and non-survivor groups are summarized in Table 2. Compared with survivors, non-survivors were significantly older with higher rates of female sex, obesity, smoking history, malignancies, CKD, DM, chronic viral hepatitis, liver cirrhosis and hepatic failure, COPD, ILD, HF, cIHD and sequalae of CVD (old cerebrovascular events), GC administration, and albumin preparation administration. Comparison of the treatment approaches between the two groups revealed that there were more patients receiving oral antivirals and the combination of oral and intravenous antivirals, immunoglobulin, and albumin preparations, in the non-survivor group than in the survivor group. We analyzed the poor prognostic factors associated with in-hospital mortality due to HZ using the Cox proportional hazards regression model (Figure 3). By multivariable analysis, over 75 years of age (hazard ratio [HR], 2.17; [95% CI, 1.53–3.06, *p* < 0.001]), liver cirrhosis and hepatic failure (HR, 6.84; [95% CI, 2.17–21.63, *p* = 0.001]), CKD (HR, 1.88; [95% CI, 1.27–2.78, *p* = 0.002]) and HF (HR, 1.70; 95% CI, 1.24–2.34, *p* = 0.001), and sequalae of CVD (HR, 1.96; [95% CI, 1.08–3.56, *p* = 0.028]) were poor prognostic factors. All results of the Cox hazard regression analysis are shown in Supplementary Table 1.

**Table 2:** Baseline clinical characteristics between survivors and non-survivors.

	**Survivor**	**Non-survivor**	***p*-value**
	***n* = 28,487**	***n* = 307**	
**Age**			
Year, Median (IQR)	71 [61, 80]	77 [67, 85]	<0.001
18–64 (%)	9,041 (31.5)	62 (20.2)	<0.001
65–74 (%)	8,034 (27.9)	61 (19.9)	0.001
75- (%)	11,672 (40.6)	184 (59.9)	<0.001
**Gender**			
Female (%)	15,202 (52.9)	148 (48.2)	0.108
**Body mass index (BMI)**			
Median (IQR)	17.8 [16.3, 19.5]	17.8 [16.3, 20.0]	0.024
−18.4 (%)	16,567 (57.6)	157 (51.1)	0.001
18.5–24.9 (%)	10,866 (37.8)	123 (40.1)	0.009
25.0- (%)	137 (0.5)	5 (1.6)	0.016
Missing data	1,177 (4.1)	22 (7.2)	
**Smoking**			
Brinkman index, Median (IQR)	0.00 [0.00, 0.00]	0.00 [0.00, 357.50]	<0.001
Smoking history (%)	6,397 (22.3)	92 (30.0)	0.007
Missing data	3,151 (11.0)	29 (9.4)	
**Length of hospital stay**			
Days, Median (IQR)	8 [8, 13]	36 [20, 61]	<0.001
**Types of herpes zoster**			
Central nervous system (%)	8,995 (31.3)	39 (12.7)	<0.001
Disseminated (%)	3,024 (10.5)	27 (8.8)	0.399
Ophthalmicus (%)	1,068 (3.7)	1 (0.3)	<0.001
Others (%)	19,655 (68.4)	250 (81.4)	<0.001
**Underlying diseases**			
No underlying disease (%)	11,064 (38.5)	17 (5.5)	<0.001
**Malignant diseases**			
All malignancies (%)	6,687 (23.3)	195 (63.5)	<0.001
Solid cancer (%)	2,197 (7.6)	86 (28.0)	<0.001
Malignant lymphoma and hematopoietic malignancies (%)	4,686 (16.3)	118 (38.4)	<0.001
**Autoimmune diseases**			
Connective tissue diseases (CTD) (%)	1,477 (5.1)	15 (4.9)	1.000
Rheumatoid arthritis (%)	750 (2.6)	7 (2.3)	0.858
Systemic lupus erythematosus (%)	278 (1.0)	2 (0.7)	1.000
Systemic vasculitis (%)	130 (0.5)	2 (0.7)	0.407
Other CTD (%)	420 (1.5)	6 (2.0)	0.466
Demyelinating diseases (%)	52 (0.2)	0 (0.0)	1.000
**Immune disorder**			
Human immunodeficiency virus infection (%)	75 (0.3)	0 (0.0)	1.000
Transplanted organ and tissue status (%)	271 (0.9)	10 (3.3)	0.001
Immunodeficiencies (%)	542 (1.9)	11 (3.6)	0.053
**Gastrointestinal and liver diseases**			
Inflammatory bowel diseases (%)	97 (0.3)	0 (0.0)	0.630
Autoimmune hepatitis (%)	27 (0.1)	1 (0.3)	0.257
Chronic viral hepatitis (%)	394 (1.4)	11 (3.6)	0.004
Liver cirrhosis and Hepatic failure (%)	29 (0.1)	4 (1.3)	<0.001
**Renal diseases**			
Chronic kidney disease (%)	580 (2.0)	33 (10.7)	<0.001
Glomerulonephritis (%)	181 (0.6)	3 (1.0)	0.449
**Endocrine and metabolic diseases**			
Diabetes mellitus (%)	4,784 (16.6)	65 (21.2)	0.038
Dyslipidemia (%)	3,083 (10.7)	18 (5.9)	0.005
Hyperuricemia (%)	1,489 (5.2)	20 (6.5)	0.299
Disorders of thyroid gland (%)	47 (0.2)	0 (0.0)	1.000
**Respiratory diseases**			
Asthma (%)	604 (2.1)	6 (2.0)	1.000
Chronic obstructive pulmonary disease (%)	151 (0.5)	5 (1.6)	0.025
Interstitial lung disease (%)	291 (1.0)	16 (5.2)	<0.001
**Cerebrovascular and cardiovascular diseases**			
Hypertension (HT) and HT related diseases (%)	6,334 (22.0)	74 (24.1)	0.406
Heart failure (%)	1,240 (4.3)	61 (19.9)	<0.001
Chronic ischemic heart disease (%)	189 (0.7)	2 0.7)	1.000
Sequelae of cerebrovascular disease (%)	529 (1.8)	14 (4.6)	0.002
**Psychiatric diseases**			
Depressive disorder (%)	939 (3.3)	11 (3.6)	0.745
**Cerebro-cardiovascular events (C-CVE)**			
Post-hospitalization onset of C-CVE (%)	369 (1.3)	16 (5.2)	<0.001
Post-hospitalization onset of CVD (%)	164 (0.6)	12 (3.9)	<0.001
Cerebral hemorrhage (%)	31 (0.1)	8 (2.6)	<0.001
Ischemic cerebrovascular diseases (%)	115 (0.4)	3 (1.0)	0.130
Precerebral arteries (%)	11 (0.0)	0 (0.0)	1.000
Cerebral arteries (%)	16 (0.1)	0 (0.0)	1.000
Unexplained (%)	88 (0.3)	3 (1.0)	0.072
Other CVDs (%)	22 (0.1)	1 (0.3)	0.217
Post-hospitalization onset of IHD (%)	209 (0.7)	4 (1.3)	0.293
**Anti-herpes zoster treatment**			
Prescription days of antivirals, days, Median (IQR)	8 [7, 8]	8 [7, 19]	<0.001
Oral drug monotherapy (%)	5,846 (20.3)	111 (36.2)	<0.001
Acyclovir (ACV) (%)	3,141 (10.9)	61 (19.9)	<0.001
Valaciclovir (VCV) (%)	2,258 (7.9)	41 (13.4)	0.001
Famciclovir (FCV) (%)	321 (1.1)	7 (2.3)	0.091
Amenamevir (ANV) (%)	26 (0.1)	0 (0.0)	1.000
Intravenous monotherapy (%)	21,257 (73.2)	151 (49.2)	<0.001
ACV (%)	21,203 (73.8)	146 (47.6)	<0.001
Combination of oral and intravenous drugs (%)	1,991 (6.9)	45 (14.7)	<0.001
ACV and ACV (%)	194 (0.7)	15 (4.9)	<0.001
VCV and ACV (%)	981 (3.4)	19 (6.2)	0.016
FCV and ACV (%)	288 (1.0)	4 (1.3)	0.556
ANV and ACV (%)	26 (0.1)	1 (0.3)	0.249
**Medications administered during hospitalization**			
Glucocorticoids (%)	9,361 (32.6)	189 (61.6)	<0.001
Albumin preparations (%)	187 (0.7)	58 (18.9)	<0.001
Globulin preparations (%)	582 (2.0)	50 (16.3)	<0.001
**Prescription at discharge for post herpetic neuralgia**			
Non-steroidal anti-inflammatory drugs (%)	6,939 (23.9)		
Voltage-gated Ca^2+^ channel α 2 δ ligand (%)	7,882 (27.1)		
Weak opioids (%)	3,016 (10.4)		
Strong opioids (%)	701 (2.4)		
Serotonin-noradrenaline reuptake inhibitor (%)	409 (1.4)		
Tricyclic antidepressant (%)	714 (2.5)		
Antiarrhythmic drugs (%)	59 (0.02)		

*ACV, acyclovir; ANV, Amenamevir; BMI, Body mass index; C-CVE, Cerebro-cardiovascular events; cIHD, Chronic ischemic heart disease; CTD, Connective tissue diseases; CVD, Cerebrovascular disease; FCV, Famciclovir; HT, Hypertension; HZ, Herpes zoster; IHD, Ischemic heart disease; VCV, Valaciclovir; VDB, Vidarabine*.

*The Mann Whitney test, Chi-square test, and Fisher's exact test were used when appropriate to compare the groups*.

#### C-CVE In-hospital Onset Rates and Risk Factors

**Figure 4:**
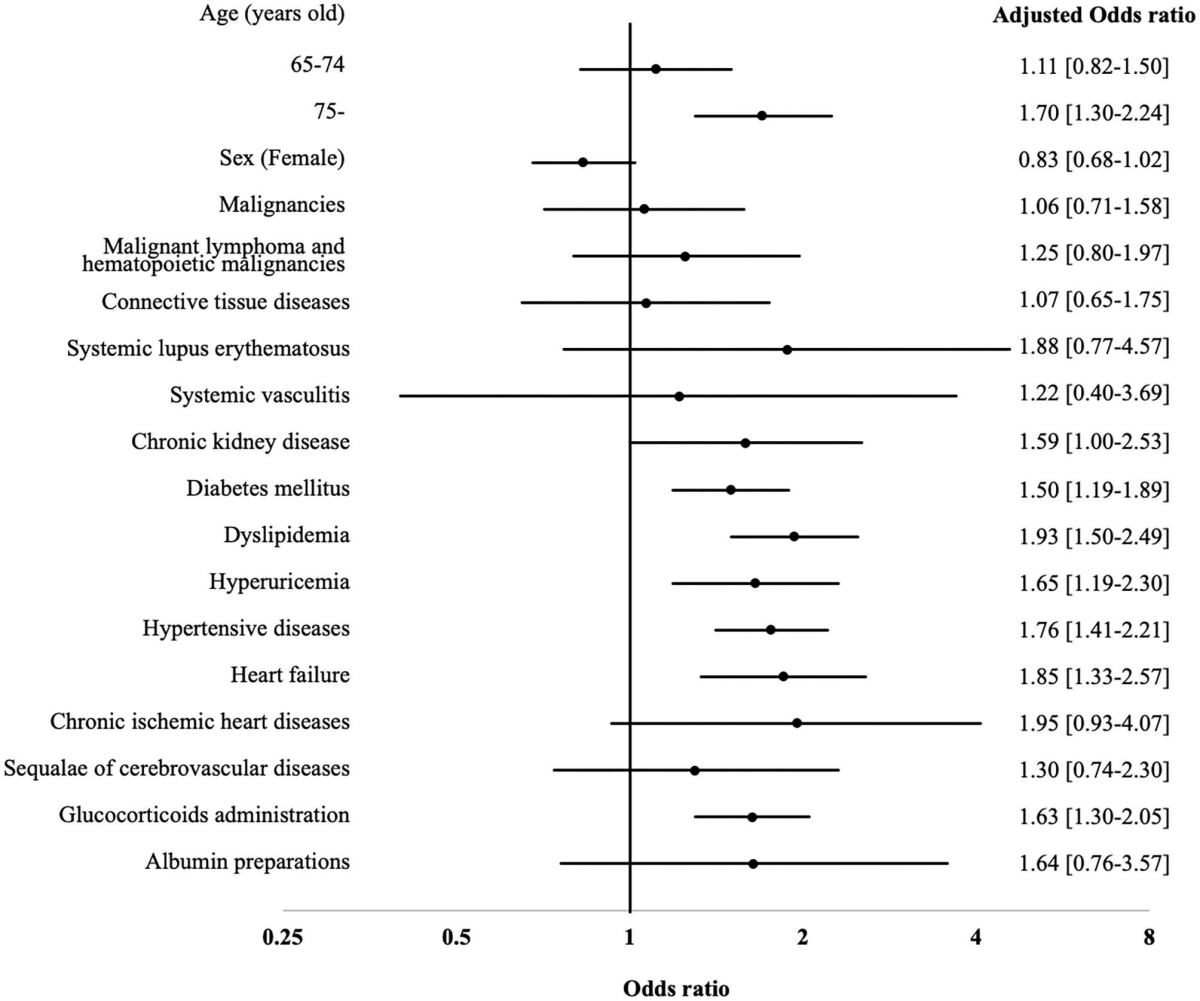
Predictors for risk factors of C-CVE onset after hospitalization. aOR, adjusted odds ratio; C-CVE, cerebro-cardiovascular events; 95% CI, 95 percent confidence interval.

Three hundred and eighty five cases (1.3%) experienced in-hospital C-CVE onset after hospitalization. The baseline characteristics of the non-C-CVE onset and C-CVE onset groups are summarized in Table 3. Compared with the non-C-CVE onset group, the C-CVE onset group were significantly older and had significantly higher rates of death, male, smoking history, malignant lymphoma and hematopoietic malignancies, transplanted organ and tissue status, CKD, DM, dyslipidemia, hyperuricemia, asthma, ILD, HT, HF, cIHD, sequalae of CVD, transplanted organ and tissue status, and GC administration during hospitalization. Comparison of treatment approaches between the two groups revealed that there were more patients receiving oral antiviral monotherapy, immunoglobulin, and albumin preparation in the C-CVE onset group than in the non-C-CVE onset group. We analyzed the risk factors associated with in-hospital C-CVE onset after hospitalization for HZ treatment. By multivariable logistic regression analysis, older age ≥ 75 years (odds ratio [OR], 1.70; [95% CI, 1.30–2.24, *p* < 0.001]), DM (OR, 1.50; [95% CI, 1.19–2.49, *p* = 0.001]), hyperuricemia (OR, 1.65; [95% CI, 1.19–2.30]), HT (OR, 1.76; [95% CI, 1.41–2.30, *p* < 0.001]), HF (OR, 1.85; [95% CI, 1.33–2.57, *p* < 0.001]), and GC administration (OR, 1.63; [95% CI, 1.30–2.05, *p* < 0.001]) were identified as risk factors for in-hospital C-CVE onset (Figure 4). The results of the logistic regression analysis are shown in Supplementary Table 2.

**Table 3:** Baseline clinical characteristics between C-CVE onset cases and no occurrence of C-CVE cases.

	**No occurrence**	**C-CVE onset**	***p*-value**
	**of C-CVE**	**after**	
	***n* = 28,669**	**hospitalization**	
		***n =* 385**	
**Age**			
Year, Median (IQR)	71 [61, 80]	76 [66, 83]	<0.001
18–64 (%)	9,020 (31.5)	83 (21.6)	<0.001
65–74 (%)	8,002 (27.9)	93 (24.2)	0.109
75- (%)	11,647 (40.6)	209 (54.3)	<0.001
**Gender**			
Female (%)	18,250 (52.0)	240 (45.1)	0.002
**Body mass index (BMI)**			
Median (IQR)	17.8 [16.3, 19.5]	18.0 [16.5, 19.7]	0.073
−18.4 (%)	16,512 (57.6)	212 (55.1)	0.258
18.5–24.9 (%)	10,836 (37.8)	153 (39.7)	0.395
25.0- (%)	142 (0.5)	0 (0.0)	0.333
Missing data	1,179 (4.1)	20 (5.2)	
**Smoking**			
Brinkman index, Median (IQR)	0.00 [0.00, 1.00]	0.00 [0.00, 80.00]	0.221
Smoking history (%)	6,401 (22.3)	88 (22.9)	0.003
Missing data	3,117 (10.9)	63 (16.4)	
**Length of hospital stay**			
Days, Median (IQR)	8 [8, 13]	12 [8, 22]	<0.001
**Types of herpes zoster**			
Central nervous system (%)	8,920 (31.1)	114 (29.6)	0.543
Disseminated (%)	3,017 (10.5)	34 (8.8)	0.315
Ophthalmicus (%)	1,057 (3.7)	12 (3.1)	0.682
Others (%)	19,636 (68.5)	269 (69.9)	0.581
**Underlying diseases**			
No underlying disease (%)	11,081 (38.7)	0 (0.0)	<0.001
**Malignant diseases**			
All malignancies (%)	6,767 (23.6)	115 (29.9)	0.005
Solid cancer (%)	2,251 (7.9)	32 (8.3)	0.703
Malignant lymphoma and hematopoietic malignancies (%)	4,717 (16.5)	87 (22.6)	0.002
**Autoimmune diseases**			
Connective tissue diseases (CTD) (%)	1,464 (5.1)	28 (7.3)	0.062
Rheumatoid arthritis (%)	745 (2.6)	12 (3.1)	0.517
Systemic lupus erythematosus (%)	273 (1.0)	7 (1.8)	0.104
Systemic vasculitis (%)	128 (0.4)	4 (1.0)	0.099
Other CTD (%)	419 (1.5)	7 (1.8)	0.518
Demyelinating diseases (%)	51 (0.2)	1 (0.3)	0.501
**Immune disorder**			
Human immunodeficiency virus infection (%)	75 (0.3)	0 (0.0)	0.629
Transplanted organ and tissue status (%)	273 (1.0)	8 (2.1)	0.035
Immunodeficiencies (%)	549 (1.9)	4 (1.0)	0.261
**Gastrointestinal and liver diseases**			
Inflammatory bowel diseases (%)	96 (0.3)	1 (0.3)	1.000
Autoimmune hepatitis (%)	28 (0.1)	0 (0.0)	1.000
Chronic viral hepatitis (%)	400 (1.4)	5 (1.3)	1.000
Liver cirrhosis and hepatic failure (%)	32 (0.1)	1 (0.3)	0.356
**Renal diseases**			
Chronic kidney disease (%)	592 (2.1)	21 (5.5)	<0.001
Glomerulonephritis (%)	182 (0.6)	2 (0.5)	1.000
**Endocrine and metabolic diseases**			
Diabetes mellitus (%)	4,741 (16.5)	108 (28.1)	<0.001
Dyslipidemia (%)	3,007 (10.5)	94 (24.4)	<0.001
Hyperuricemia (%)	1459 (5.1)	50 (13.0)	<0.001
Disorders of thyroid gland (%)	47 (0.2)	0 (0.0)	1.000
**Respiratory diseases**			
Asthma (%)	596 (2.1)	14 (3.6)	0.046
Chronic obstructive pulmonary disease (%)	153 (0.5)	3 (0.8)	0.466
Interstitial lung disease (%)	298 (1.0)	9 (2.3)	0.022
**Cerebrovascular and cardiovascular diseases**			
Hypertension (HT) and HT related diseases (%)	6,246 (21.8)	162 (42.1)	<0.001
Heart failure (%)	1,256 (4.4)	45 (11.7)	<0.001
Chronic ischemic heart disease (%)	183 (0.6)	8 (2.1)	0.004
Sequelae of cerebrovascular disease (%)	530 (1.8)	13 (3.4)	0.036
**Psychiatric diseases**			
Depressive disorder (%)	933 (3.3)	17 (4.4)	0.194
In-hospital Death (%)	291 (1.0)	16 (4.2)	<0.001
**Breakdown of C-CVE**			
Post-hospitalization onset of CVD (%)		176 (45.8)	
Cerebral hemorrhage (%)		39 (10.1)	
Ischemic cerebrovascular diseases (%)		118 (30.6)	
Precerebral arteries (%)		11 (2.9)	
Cerebral arteries (%)		16 (4.2)	
Unexplained (%)		91 (23.6)	
Other CVDs (%)		23 (6.0)	
Post-hospitalization onset of IHD (%)		213 (55.3)	
**Anti-herpes zoster treatment**			
Prescription days of antivirals, days, Median (IQR)	8 [7, 8]	8 [7, 12]	<0.001
Oral drug monotherapy (%)	5,853 (20.4)	104 (27.0)	0.002
Acyclovir (ACV) (%)	3,137 (10.9)	65 (16.9)	0.001
Valaciclovir (VCV) (%)	2,266 (7.9)	33 (8.6)	0.634
Famciclovir (FCV) (%)	324 (1.1)	4 (1.0)	1.000
Amenamevir (ANV) (%)	26 (0.1)	0 (0.0)	1.000
Intravenous monotherapy (%)	20,807 (72.6)	254 (66.0)	0.005
ACV (%)	21,095 (73.6)	254 (66.0)	0.001
Combination of oral and intravenous drugs (%)	2,009 (7.0)	27 (7.0)	1.000
ACV and ACV (%)	205 (0.7)	4 (1.0)	0.362
VCV and ACV (%)	988 (3.4)	12 (3.1)	0.888
FCV and ACV (%)	290 (1.0)	2 (0.5)	0.600
ANV and ACV (%)	26 (0.1)	1 (0.3)	0.303
**Medications administered during hospitalization**			
Glucocorticoids (%)	9,380 (32.7)	170 (44.2)	<0.001
Albumin preparations (%)	238 (0.8)	7 (1.8)	0.046
Globulin preparations (%)	619 (2.2)	13 (3.4)	0.111
**Prescription at discharge for post herpetic neuralgia**			
Non-steroidal anti-inflammatory drugs (%)	5,525 (19.3)	66 (17.1)	0.329
Voltage-gated Ca^2+^ channel α 2 δ ligand (%)	6,401 (22.3)	69 (17.9)	0.042
Weak opioids (%)	2,316 (8.1)	34 (8.8)	0.572
Strong opioids (%)	271 (0.9)	8 (2.1)	0.033
Serotonin-noradrenaline reuptake inhibitor (%)	244 (0.9)	7 (1.8)	0.051
Tricyclic antidepressant (%)	651 (2.3)	8 (2.1)	1.000
Antiarrhythmic drugs (%)	38 (0.1)	0 (0.0)	1.000

*ACV, acyclovir; ANV, Amenamevir; BMI, Body mass index; C-CVE, Cerebro-cardiovascular events; cIHD, Chronic ischemic heart disease; CTD, Connective tissue diseases; CVD, Cerebrovascular disease; FCV, Famciclovir; HT, Hypertension; HZ, Herpes zoster; IHD, Ischemic heart disease; VCV, Valaciclovir; VDB, Vidarabine*.

*The Mann Whitney test, Chi-square test, and Fisher's exact test were used when appropriate to compare the groups*.

## Discussion

### Brief Summary of This Study

We investigated short-term in-hospital mortality and incidence of C-CVE in 29,054 hospitalized cases with HZ and analyzed prognostic factors and risk factors for C-CVE onset after HZ. Over 75 years of age, cirrhosis, liver failure, CKD, HF, and sequelae of CVD were identified as short-term prognostic factors. Traditional C-CVE risk factors such as age ≥75 years, DM, dyslipidemia, hyperuricemia, HT, and HF were identified as risk factors for C-CVE development after hospitalization for HZ treatment, and GC administration was also identified as a new C-CVE risk factor.

### Interpretation

The in-hospital mortality for HZ was similar to that reported in the study by Esteban-Vasallo et al. from Spain, conducted between 2003 and 2013. The study included data from the years before the widespread use of recombinant zoster vaccine (RZV), biologics, and JAK-I (32). Since there was no substantial discrepancy in the in-hospital mortality in our study, conducted using data from 2016 to 2018, the period when immunosuppressive therapy was developed, it is conceivable that biologics and JAK-i could have an impact on the development of HZ but might not have a significant impact on the severity of HZ. The incidence of HZ is higher in Japanese RA patients receiving JAK-i, but there may not be as much racial difference in mortality after HZ onset as HZ incidence in the unvaccinated population (5, 10, 33). Cirrhosis and liver failure, CKD, HF, and old cerebrovascular events, identified as risk factors for in-hospital mortality in this study, are also considered to be risk factors for severity of other viral infections such as influenza (34). HZ vaccines have been reported to be effective in preventing the onset and severity of HZ (35). For the prevention of severe conditions, the priority of vaccination might be based on the same criteria as that for vaccinations against other viral infections such as influenza because HZ is a common disease. It is important to address the prevention of HZ because if the onset and severity of HZ can be prevented, the complications of C-CVE might also be reduced. However, there are still many unclear points, such as the duration of the vaccine effects, therefore, further studies are needed on the timing of vaccination and the selection of priority vaccination targets.

Even in severe HZ, it was suggested that an inflammatory response (vasculitis) to the virus may occur from the acute stage after HZ onset, leading to the development of C-CVE. Inflammatory reactions in the artery and endothelial dysfunction are involved in the development of C-CVE (36, 37). Therefore, patients who have an underlying disease with arteriosclerosis and/or endothelial dysfunction such as diabetes may need to be especially attentive to the development of C-CVE after HZ. In addition to risk factors such as DM and HT, GC administration was newly identified as a risk factor for C-CVE onset after onset of HZ. GC has been previously reported to be a risk factor for C-CVE in CTD (38–40). GC administration may be used as adjunctive therapy for Ramsay-Hunt syndrome or for pain relief and prevention of sequelae of HZ, but administration of GC should be avoided because it may increase the risk of C-CVE (41). Since CTD, which requires a relatively frequent administration of GC, was not identified as a risk factor for C-CVE onset, it is possible that GC administration itself may lead to the C-CVE risk after HZ onset. In patients with a disease which is needed for long-term GC administration such as CTD, it would be desirable to reduce or avoid GC administration as much as possible to prevent HZ development and C-CVE onset after HZ.

### Strengths of This Study

The strength of this study lies in the large sample size. We analyzed over 29,000 hospitalized HZ cases, the largest numbers ever studied. In previous studies, it could have been difficult to detect HZ-associated deaths and C-CVE onset associated with HZ during hospitalization due to the relatively low patient numbers. We were able to detect more than 300 cases of both in-hospital death and C-CVE onset, which allowed us to conduct analyses on short-term prognostic factors and risk factors for C-CVE onset.

In addition, the DPC database we used covers many acute care hospitals, including advanced care hospitals such as university hospitals, contributing to the generalization and comprehensiveness of the analysis results (42). Another strength of this study is that it covers a relatively small number of underlying diseases, such as CTD. Because relatively rare underlying diseases were included in the analysis, a more comprehensive analysis was possible.

### Limitations

This study has several limitations. First, laboratory test results, imaging findings, and medical records were not available from the DPC database. Therefore, the diagnosis may uncertain. However, the diagnostic accuracy of DPC is moderate or high (43, 44), and previous studies using database have also used the ICD codes (25, 32). We used information on antivirals for HZ to improve diagnostic accuracy. Since antivirals for HZ are not administered for other infections, patients administered with antivirals for more than a certain period are more likely to be true HZ. Second, the DPC database contains no information before hospitalization, such as prescription drugs or HZ vaccination, and post-discharge outcomes. Since there is a possibility that the risk of HZ-related death and C-CVE may increase after discharge from the hospital, a long-term investigation should be conducted. The effect of vaccination on this study could be limited. Few people were vaccinated at the time of this analysis, because ZVL (zoster vaccine live) and RZV were approved in 2016 and 2018, respectively, for those over 50 years in Japan. ZVL is contraindicated during immunosuppressive and anticancer chemotherapy in Japan. Many patients with underlying diseases, such as CTD or malignancies, were not vaccinated. Future studies should examine any change in mortality and C-CVE incidence with vaccination. These limitations can be overcome by matching DPC data with the National Database of Health Insurance Claims data, which contain information on prescription drugs in outpatient settings. However, there are many institutional and technical problems in linking the two databases. If institutional changes and technological innovations permit us to link the databases before and during hospitalization, we would like to investigate the linking of individuals' data.

## Conclusion

Aggressive HZ prevention, including vaccination, should be considered for patients older than 75 years and for patients with poor prognostic factors. In addition to the conventional C-CVE risk factors, GC might be a risk factor for the development of C-CVE after severe HZ onset.

## Data Availability Statement

YI, KT, and KF had full access to all of the data in the study and take responsibility for the integrity of the data and the accuracy of the data analysis. In this study, we used the DPC data provided by acute care hospitals in Japan to the DPC Research Institute. We conducted our study using this DPC database at the University of Occupational and Environmental Health, Japan. This database can only be accessed for research purpose.

## Ethics Statement

Informed consent was waived for all patients included in this retrospective cohort study, and all information extracted was anonymized. The Institutional Review Board of the University of Occupational and Environmental Health, Japan approved this study (approval code: R2-007). Written informed consent for participation was not required for this study in accordance with the national legislation and the institutional requirements.

## Author Contributions

YI, KT, and KN: conceptualization and design, acquisition, analysis, and interpretation of data. KF: acquisition, analysis and interpretation of data. YI: drafting of the manuscript and statistical analysis. KN, SN, SM, and YT: supervision. All authors: critical revision of the manuscript for important intellectual content. All authors contributed to the article and approved the submitted version.

## Conflict of Interest

KN has received speaking fees from Sanofi, AbbVie, Eisai, Eli Lilly, Chugai, Pfizer, GSK, Astellas, UCB, and Mitsubishi-Tanabe, and research grants from Boehringer Ingelheim, Chugai, Eisai, AbbVie, Asahi Kasei, Ayumi, Eli Lilly, and UCB. SN has received consulting fees, speaking fees, and/or honoraria from Bristol-Myers, Pfizer, GlaxoSmithKline, Sanofi, Astellas, Asahi-Kasei, and Boehringer Ingelheim and has received research grants from Mitsubishi-Tanabe and Novartis. YT has received speaking fees and/or honoraria from Gilead, Abbvie, Behringer-Ingelheim, Eli Lilly, Mitsubishi-Tanabe, Chugai, Amgen, YL Biologics, Eisai, Astellas, Bristol-Myers, and Astra-Zeneca; received research grants from Asahi-Kasei, Abbvie, Chugai, Mitsubishi-Tanabe, Eisai, Takeda, Corrona, Daiichi-Sankyo, Kowa, and Behringer-Ingelheim; and received consultant fees from Eli Lilly, Daiichi-Sankyo, Taisho, Ayumi, Sanofi, GSK, and Abbvie. The remaining authors declare that the research was conducted in the absence of any commercial or financial relationships that could be construed as a potential conflict of interest.

## Publisher's Note

All claims expressed in this article are solely those of the authors and do not necessarily represent those of their affiliated organizations, or those of the publisher, the editors and the reviewers. Any product that may be evaluated in this article, or claim that may be made by its manufacturer, is not guaranteed or endorsed by the publisher.
